# Immunohistochemical Features of Breast Cancer Seen in Women in Kinshasa, Democratic Republic of the Congo: A Six-Year Retrospective Study

**DOI:** 10.1155/2022/8860947

**Published:** 2022-08-05

**Authors:** Stanislas Maseb'a Mwang Sulu, Donatien Babaka Batalansi, Arnold Maseb Sul Sulu, Olivier Mukuku, Justin Esimo Mboloko, Désiré Kulimba Mashinda, Bienvenu Lebwaze Massamba, Antoine Wola Tshimpi

**Affiliations:** ^1^Nganda Hospital Center, Kinshasa, Democratic Republic of the Congo; ^2^Kinshasa Provincial General Reference Hospital, Kinshasa, Democratic Republic of the Congo; ^3^Institut Supérieur des Techniques Médicales de Lubumbashi, Lubumbashi, Democratic Republic of the Congo; ^4^Kinshasa University Clinics, Kinshasa, Democratic Republic of the Congo; ^5^School of Public Health, University of Kinshasa, Kinshasa, Democratic Republic of the Congo

## Abstract

**Introduction:**

The molecular classification of breast cancer (BC) based on gene expression and then protein profile has made it possible to distinguish different molecular subtypes. The objective of this study was to describe immunohistochemical features of BC infiltrating women at the Nganda Hospital Center in Kinshasa, Democratic Republic of the Congo (DRC).

**Methods:**

A retrospective study from 2014 to 2019 involved 190 patients with invasive BC who were enrolled at the Nganda Hospital Center. The tumors were analyzed histologically and classified after an immunohistochemical study into subtypes: luminal A, luminal B, HER2-overexpressed, and triple-negative. A chi-square test was performed to assess the relationship between age, histological grade, and these subtypes.

**Results:**

The luminal A subtype (44.74%) was the most common, followed by luminal B (40.53%), triple-negative (10.53%), and HER2-overexpressed (4.20%). The mean age of the patients at diagnosis was 48.27 years. Of all cases, 94.21% were ductal, 2.63% were mucinous, and 2.11% were lobular. They were classified as grade I in 68.82% of the cases, grade II in 28.42% of the cases, and grade III in 3.16% of the cases. There was a significant association between histological grade and breast cancer subtypes (*p* < 0.0001), but no correlation was found with age (*p* = 0.467).

**Conclusion:**

In our BC patients, the luminal A was predominant, while HER2-overexpressed was the least found. A strong association was noted between histological grade and molecular subtypes. These results should allow for important clinical and policy implications for BC control in the DRC.

## 1. Introduction

Breast cancer (BC) is a major public health problem with an increasing trend in prevalence and mortality rates worldwide [1]. By 2020, according to recent estimates by the World Health Organization (WHO), 2.3 million women with BC and 685,000 deaths from BC had been recorded worldwide [2]. In sub-Saharan Africa (SSA), according to recent estimates, the overall combined gross incidence rate was estimated at 24.0 per 100,000 person-years (95% confidence interval (95% CI): 17.5-30.4) in hospital and 22.4 per 100,000 (95% CI: 17.2-28.0) in community [3]. The BC diagnosis is frequently made between the ages of 35 and 49 among African women, fifteen years sooner than European and North American women [4]. Furthermore, the mortality rate among SSA women is higher, as tumors are more aggressive and the time between the onset of symptoms and diagnosis is shorter [5].

Few studies on BC have previously been conducted in the DRC [6]. During the 10-year period from June 2010 to June 2020, 5801 cases of cancer (all sexes) were recorded in five pathological laboratories in Kinshasa. Katumbayi et al. [7] had 3163 cancers in women, of which BC was predominant (49.9%). Another study by Mbala in Kinshasa, in a series of 50 patients, showed that estrogenic and progesteronic receptors were detected in 86% and overexpression of the human epidermal growth factor 2 (HER2) receptor was absent in 90% of cases [8]. BC is a heterogeneous disease, varying from clinical presentation to molecular characteristics, and tends to present a potentially distinct prognosis [9]. Evaluation of HER2 overexpression and immunohistochemical determination of estrogen and progesterone receptors identify the different molecular subtypes of BC. These subtypes include luminal A, luminal B, HER2-overexpressed, and triple negative. The last two subtypes are hormone receptor negative and have a poor prognosis [10, 11]. This immunohistochemical assessment of BC is useful in defining different prognostic subgroups with different relationships to the adjuvant treatment received [12].

The objective of this study was to describe the main immunohistochemical features of BC in the oncology department of the Nganda Hospital Center in Kinshasa, DRC.

## 2. Materials and Methods

### 2.1. Study Framework and Period

This was a retrospective study of histologically confirmed cases of BC in the oncology department of the Nganda Hospital Center in Kinshasa (DRC) for BC over a period of 6 years from January 2014 to December 2019.

Nganda Hospital Center is one of the health facilities in the DRC specialized in cancer management. It is composed of one oncologist, two radiation oncologists, and one pathologist. This center has a laboratory for pathology and immunohistochemistry and a radiotherapy service that serves not only the city of Kinshasa but also the entire DRC and some neighboring countries.

### 2.2. Study Population and Immunohistochemical Analyses

We included in this study 190 women with invasive BC and complete immunohistochemical analyses. Excluded from this study were all patients with a BC whose records were unusable. Age and histological data were collected from anatomopathological reports (histological type, Scarff-Bloom-Richardson histoprognostic grade modified by Elston and Ellis) and also from immunohistochemical reports (assay of estrogen receptor (ER), progesterone receptor (PgR), HER2-overexpressed, and Ki-67 levels). The tissues were previously fixed in a 10% formalin solution. For each of the 190 patients selected, paraffin inclusion blocks were collected. Histological sections 3-5 *μ*m thick were stained with hematoxylin-eosin for the morphological study. Immunohistochemistry techniques were carried out, using the three-layer immunoperoxidase technique, for the detection of the expression of hormone receptors (ER and PgR) and of the proliferation index Ki-67. The overexpression of the oncogene HER2 was also investigated. The study method included a first histological analysis for the selection of antibodies for immunolabeling. A second reading with immunohistochemistry slides was carried out, and a third reading was carried out as an internal quality control to reach a consensus diagnosis for each case, based on the WHO classification revised in 2019. The tumor sections were incubated with the specific primary mouse monoclonal antibodies against the ER (clone 1D5, dilution 1: 100; Dako, Glostrup, Denmark) and PgR (clone 1A6, dilution 1: 800; Dako); the Ki-67 antigen was evaluated using the monoclonal antibody MIB1 (dilution 1: 200; Dako), and HER2 was evaluated using Dako polyclonal antibody (dilution 1: 1600). The percentages of neoplastic cells with a defined nuclear immunoreactivity among 2000 cells in at least 10 randomly selected high potency (400x magnification) fields were recorded for ER, PgR, and Ki-67. For the HER2 assessment, tumors were scored according to the intensity and completeness of cell membrane staining on a 4-level scale (0: no immunoreactivity, 1+: weak and incomplete membrane staining, 2+: weak/moderate and complete membrane staining, and 3+: strong and complete membrane staining); the percentage of immunoreactive neoplastic cells was also recorded. The 3+ tumors were considered for HER2-overexpressed. The positivity threshold for ER and PgR was 1%, and the positivity threshold for MIB1 was 20%, as described in the literature [13]. The immunohistochemical classification was as follows [10]:

- Luminal A (ER + or PgR ±) and (Ki − 67 < 14%) and (HER2 -)
- Luminal B (ER + or PgR ±) and ((Ki − 67 ≥ 14%) or (HER2 ±))
- HER2-overexpressed (ER - and PgR -) and (HER2 +)
- Triple negative (ER - and PgR -) and (HER2 -)

### 2.3. Statistical Analyses

Data were analyzed using the Stata 16 software. The synthesis of the modalities of the qualitative variables was in the form of frequencies. For quantitative variables, the synthesis was calculated by means and standard deviations. Correlations between age groups, histological grade, and subtypes of BC were measured in contingency tables using chi-squared association tests. In addition, the ANOVA test was used to determine whether the mean age values were different between subtypes of BC. A value of *p* < 0.05 was considered significant.

## 3. Results

The mean age of the patients was 48.27 ± 10.33 years (range: 26 and 75 years); 37 patients (19.47%) were under 40 years of age, and 79 patients (41.58%) were 50 years of age or older.

The tumor involved the right breast in 105 patients (55.26%) and the left breast in 85 patients (44.74%). The predominant histological subtype of invasive BC was ductal carcinoma (94.21%). Mucinous carcinoma represented 2.63% and lobular carcinoma 2.11%. According to the Elston and Ellis histoprognostic grade, tumors were classified as grade I in 130 patients (68.42%), grade II in 54 patients (28.42%), and grade III in 6 patients (3.16%) (Table 1).

**Figure 1:**
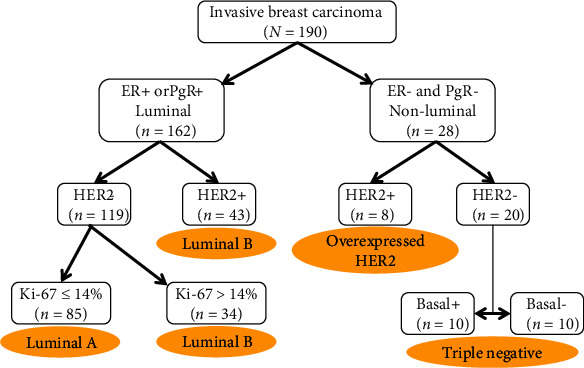
Classification of breast cancer subtypes by immunohistochemical marker profile.

The immunohistochemical study showed that invasive BC was ER positive in 162 patients (85.26%), PgR positive in 147 patients (77.37%), and HER2-overexpressed in 51 patients (26.84%). Ninety-eight patients (51.58%) had a Ki-67 of less than 14%. Consequently, 85 BC (44.74%) were classified as luminal A, 77 (40.53%) as luminal B, 8 (4.21%) as HER2-overexpressed, and 20 (10.53%) as triple negative (Figure 1).

**Figure 2:**
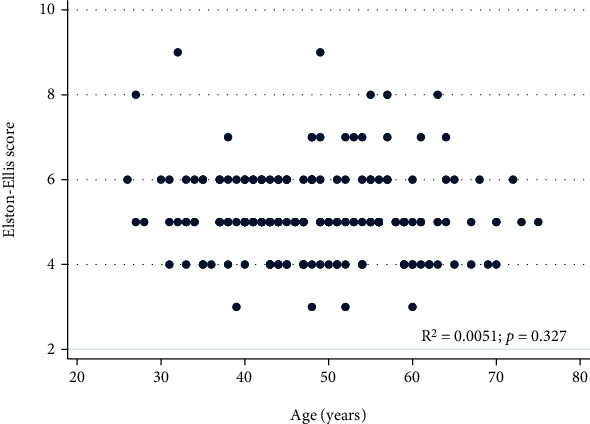
Correlation of Elston-Ellis score and age.

Elston-Ellis score did not vary with age (*p* = 0.327). Analysis of the correlation between Elston-Ellis score and age is illustrated in Figure 2.

The luminal A (47.03 years) and HER2-overexpressed (47.87 years) subtypes had the lowest mean diagnostic ages compared to the luminal B (49.50 years) and triple negative (48.95 years) subtypes; the ANOVA test does not show any significant difference between these different mean ages (*p* = 0.494). Almost one in two patients in the luminal B group (49.35%) had been diagnosed after 49 years, while 75% of the patients in the HER2-overexpressed group were less than 50 years at the time of diagnosis. The age distribution at diagnosis by subtype is presented in Table 2.

Twenty percent of triple-negative cancers were grade III, while those in luminal A were 77.65% grade I. There was a significant association between histological grade and subtypes of BC (*p* < 0.0001), but no correlation was found with age (*p* = 0.467) (Table 2).

## 4. Discussion

The purpose of this study was to provide a contribution by establishing an immunohistochemical profile of BC that could assist in the effective management of patients with BC that require knowledge of hormone receptor status and HER2-overexpressed.

The mean age at diagnosis was 48.27 ± 10.33 years (range: 26 and 75 years); 58.42% of the patients were under 50 years of age. Another Congolese study carried out among 430 women with BC in 3 hospitals in the city of Kinshasa (Saint Joseph Hospital, Kinshasa General Provincial Reference Hospital, and Nganda Hospital Center) during the period from 1 January 2005 to 31 December 2015 reported a mean age of 48.5 ± 10.2 years [14]. A Cameroonian study found a mean age of 47.83 ± 13.57 years [15]. In this study, the mean age is consistent with the mean age at diagnosis of BC (43-49 years) as reported in several SSA studies [16–20]. Unlike in the SSA countries, the mean age at diagnosis in most Western series is around 60 years [21]. This is explained, in part, by the reversal of age pyramids in SSA including the DRC, with a younger population compared to developed countries [22].

The present study is the third in the DRC to determine the molecular subtypes of BC based on the immunohistochemical expression of ER, PgR, and HER2. In this study, positive hormone receptor immunostaining was found in 85.26% of the cases (85.26% for ER and 77.37% for PgR), and HER2 was positive in 26.84% of the cases. The first Congolese study, carried out more than a decade ago with 50 women with BC, reported that ER and PgR were detected in 86% and overexpression of HER2 was absent in 90% of cases [8]. In another series of 87 women with BC in Kinshasa, the second Congolese study reported that ER, PgR, and HER2 were positive at 59.8%, 65.4%, and 23%, respectively [23].

A recent systematic review and meta-analysis by Eng et al. [24] reported that most cases of BC in Africa were positive for ER. As in our study, Adani-Ifè et al. [25] found that the proportion of patients expressing ER (54.7%) was higher than that of PgR positive (41%). The same observation has been reported by different authors [18, 26–28]. Nevertheless, the positivity of hormone receptors in BC remains varied and heterogeneous across countries. In most studies of the BC distribution, luminal subtype A was found to be the most widespread [18, 19, 26–28]. However, in a series of 114 Tunisian women with invasive BC, Mahjoub et al. [29] found that luminal B was predominant (32.5% of cases), followed by the HER2-overexpressed group (26.3% of cases), luminal A group (25.4%), and triple negative (15.8%). In contrast to our study, approximately half of the cases (52.8%) in the Saudi study of Al-Tamimi et al. [30] were found to be triple negative, while luminal tumors accounted for 28.5%. Fitzpatrick et al. [16], in a series of 197 Senegalese women with BC, had 46.7% triple negative molecular subtype. Our results are contrary to those of Atangana et al. [15] who had shown that the most common subtype was triple negative (37.98%), followed by luminal A tumors (36.06%), HER2-overexpressed tumors (12.98%), and luminal B tumors (7.21%). Other SSA studies had also found a predominance of the triple negative phenotype [25, 31]. Triple negative is considered to be more common in younger women and is associated with aggressive clinicopathological characteristics [32, 33]. Our findings do not support these reports.

According to Al-Thoubaity [28], any minor geographic variation in the proportions of tumor subtypes could be related to environmental factors, genetic factors, and/or technological disparity. According to Atangana et al. [15], the high frequency of triple negative phenotype in black populations may also be related to the high frequency of BRCA1 and BRCA2 mutations in this population.

HER2-positive was observed in 26.84% of our patients. This result is slightly higher than the literature data which reported 15-20% of HER2-positive in invasive BC [34]. Our result is similar to that reported for Ghanaian women (25.5%) [35].

In this study, there was no significant difference between molecular subtypes and age (*p* = 0.467), as observed in other studies [15, 36]. However, a few studies have found a significant correlation between age and subtype of BC [19, 37]. BC subtypes were correlated with histological grade (*p* < 0.0001). A significant association between the histological grade and the molecular subtype of BC was also reported in Ivorian [18], Togolese [25], Moroccan [38], Sudanese, and Eritrean women [39].

The limitations of this study include retrospectiveness and selection bias due to the recruitment of immunohistochemically screened participants and lack of clinicopathological information; this has affected the representativeness of our sample. Larger studies from population-based samples are needed to help guide BC control programs. Despite its limitations, our study reinforces the idea that investments in the use of hormonal and anti-HER2 therapies have the potential to impact survival in Congolese patients with BC.

## 5. Conclusion

In our BC patients, the luminal A was predominant, while HER2-overexpressed was least found. A strong association between histological grade and molecular subtypes has been noted. These results are expected to have important clinical and policy implications for the control of BC in the DRC.

## Figures and Tables

**Table 1:** Age, pathological, and immunohistochemical characteristics of breast cancer.

Variable	Number (*n* = 190)	Percentage
Age		
<40 years	37	19.47
40-49 years	74	38.95
≥50 years	79	41.58
Mean ± SD	48.27 ± 10.33	
Elston and Ellis histoprognostic grade		
I	130	68.42
II	54	28.42
III	6	3.16
Histological subtype		
Ductal	179	94.21
Mucinous	5	2.63
Lobular	4	2.11
Apocrine	1	0.53
Papillary	1	0.53
Estrogen receptors		
Negative	28	14.74
Positive	162	85.26
Progesterone receptors		
Negative	43	22.63
Positive	147	77.37
HER2		
Negative	139	73.16
Positive	51	26.84
Ki-67		
≤14%	98	51.58
>14%	92	48.42

**Table 2:** Age and histoprognostic grade by breast cancer subtypes.

Variable	Luminal A *N* = 85 *n* (%)	Luminal B *N* = 77 *n* (%)	HER2 + *N* = 8 *n* (%)	Triple negative *N* = 20 *n* (%)	*p* value
Age					0.467
< 40 years	20 (23.53)	13 (16.88)	2 (25.00)	2 (10.00)	
40-49 years	35 (41.18)	26 (33.77)	4 (50.00)	9 (45.00)	
≥ 50 years	30 (35.29)	38 (49.35)	2 (25.00)	9 (45.00)	
Mean ± SD	47.03 ± 10.74	49.50 ± 9.96	47.87 ± 13.49	48.95 ± 8.67	0.494^∗^
Grade					<0.0001
I	66 (77.65)	52 (67.53)	4 (50.00)	8 (40.00)	
II	19 (22.35)	23 (29.87)	4 (50.00)	8 (40.00)	
III	0 (0.00)	2 (2.60)	0 (0.00)	4 (20.00)	

^∗^ANOVA test.

## Data Availability

The datasheet used to support the findings of this study is available from the corresponding author upon request.

## Abbreviations

95% CI: 95% confidence interval
BC: Breast cancer
DRC: Democratic Republic of the Congo
ER: Estrogen receptors
HER2: Human epidermal growth factor 2 receptor
PgR: Progesterone receptors
WHO: World Health Organization.
